# In-Field Implementation of a Recombinant Factor C Assay for the Detection of Lipopolysaccharide as a Biomarker of Extant Life within Glacial Environments

**DOI:** 10.3390/bios2010083

**Published:** 2012-03-09

**Authors:** Megan J. Barnett, Jemma L. Wadham, Miriam Jackson, David C. Cullen

**Affiliations:** 1 Cranfield Health, Vincent Building, Cranfield University, Cranfield, Bedfordshire, MK43 0AL, UK; E-Mail: d.cullen@cranfield.ac.uk; 2 Bristol Glaciology Centre, School of Geographical Sciences, University of Bristol, Bristol, BS8 1SS, UK; E-Mail: j.l.wadham@bristol.ac.uk; 3 Section for Glaciers, Ice and Snow, Hydrology Department, Norwegian Water Resources & Energy Directorate, P.O. Box 5091 Maj., N-0301 Oslo, Norway; E-Mail: mja@nve.no

**Keywords:** lipopolysaccharide, endotoxin, pyrogen, portable life detection, recombinant Factor C, cryosphere, subglacial

## Abstract

The discovery over the past two decades of viable microbial communities within glaciers has promoted interest in the role of glaciers and ice sheets (the cryosphere) as contributors to subglacial erosion, global biodiversity, and in regulating global biogeochemical cycles. *In situ* or in-field detection and characterisation of microbial communities is becoming recognised as an important approach to improve our understanding of such communities. Within this context we demonstrate, for the first time, the ability to detect Gram-negative bacteria in glacial field-environments (including subglacial environments) via the detection of lipopolysaccharide (LPS); an important component of Gram-negative bacterial cell walls. In-field measurements were performed using the recently commercialised PyroGene^®^ recombinant Factor C (rFC) endotoxin detection system and used in conjunction with a handheld fluorometer to measure the fluorescent endpoint of the assay. Twenty-seven glacial samples were collected from the surface, bed and terminus of a low-biomass Arctic valley glacier (Engabreen, Northern Norway), and were analysed in a field laboratory using the rFC assay. Sixteen of these samples returned positive LPS detection. This work demonstrates that LPS detection via rFC assay is a viable in-field method and is expected to be a useful proxy for microbial cell concentrations in low biomass environments.

## 1. Introduction

The ability to quickly quantify microbial populations at a sampling site has powerful applications across many areas of science and industry. The development of rapid in-field detection methods for generic microbial populations in both food and health industries has been made possible by the detection of biomarkers, *i.e.*, chemical markers unique to extant life [1,2,3]. The best known example of such an approach is the detection of the generic biomarker adenosine triphosphate (ATP) using off-the-shelf ATP bioluminescence kits [4].

In the environmental sciences, sample analysis at the sampling site or in a field laboratory offers many advantages. These include: (i) reducing sources of error by removing the need to modify samples for transport and extended storage; (ii) collecting further samples to clarify unexpected results, and enabling the in-field development and refinement of (iii) protocols; (iv) sampling strategies and (v) hypotheses. Together these advantages offer reduced environmental impact and reduced resource usage, for a given scientific return, when compared to the well established approach of sample collection, transport and subsequent analysis in institutional laboratories. Finally, obtaining results in the field provides the opportunity for the completion of unique experiments not achievable by conventional approaches e.g., improved spatial and temporal resolution normally restricted by the logistics of sample transportation.

Icy ecosystems, in particular glaciers, are now known to be viable habitats for microbial life [5,6,7,8,9,10], yet little is known about microbial abundances or their relevance to biogeochemical processes and therefore to global biogeochemical cycles. Due to the rapidly changing nature and the remoteness of many icy environments, they provide a prime example of where the development of in-field measurements is paramount [11]. One approach to address the difficulty of collecting microbiological data is to detect biomarkers (proxies for microbial abundance, activity and population diversity), as many of these biomarkers can be detected by simple assays. In order to apply these techniques to in-field biomarker detection and quantification in glacial samples they have to be compatible with environments that are remote, hostile, operationally difficult, and often have low biomass [12]. Within the current work, we focus on the detection of lipopolysaccharide (LPS), as a biomarker, in samples from a low biomass Arctic glacier. LPS is an essential component of the cell wall of Gram-negative bacteria and, due to the common abundance of Gram-negative bacteria within the total prokaryote population, has been used as a proxy for extant microbial life [13]. Gram-negative bacteria are widely found in icy environments having been identified in glacial samples from New Zealand [7] and China [14]; in Arctic and Antarctic pack ice [15]; in Antarctic lake ice [16] and in snow from Svalbard [17]. Thus they are likely to be present in most glacial environments.

Established techniques for the quantification of LPS, or endotoxins, exist in the pharmaceutical industry as these compounds are potentially toxic if they enter the blood stream [18,19]. The terms LPS and endotoxin are often used interchangeably in the literature, but in this work we use the term LPS. Currently the standard method for the determination of LPS concentration is the *Limulus* amoebocyte lysate (LAL) assay. The LAL assay uses the hemolymph of horseshoe crabs (*Limulus*) which naturally clots in the presence of LPS. Commercial preparations of the hemolymph react to the presence of LPS producing various types of optically readable endpoints. The LAL assay has been used as a proxy for microbial concentration in various natural environments, particularly where biomass is low: hot springs in the High Arctic [20], marine sediments [13], marine water profiles [21] and in Subglacial Lake Vostok accretion ice [22]. The standard LAL assay is relatively complex, requiring skilled operators and normally requiring multiple stages of analysis and therefore has been performed in established institutional laboratories on collected ice and water samples [21,22] and onboard a research vessel [13]. In-field detection of LPS was conducted by Steele *et al.* [20] where a commercially available microfluidic LAL-based LPS detection system was used (Endosafe^®^-PTS*™*, Charles River, MA, USA). Although this system is applicable to field use, being portable and rapid, the system has been developed for pharmaceutical use and is not flexible in terms of sample type and data analysis, thus making it difficult for development with environmental applications. The preceding work has shown the potential of LPS as a proxy for microbial abundance in low biomass environments, e.g., the detection of <10**^4^** cells per mL [22]. Here we build on this work by demonstrating the detection of LPS in the field in a glacial environment by the use of a recently released commercial product, the Cambrex PyroGene^®^ recombinant Factor C LPS detection system. Briefly, the LPS activated protein (Factor C) from the LAL clotting cascade, has been isolated [23] and a recombinant form produced. This recombinant Factor C (rFC) has subsequently been developed into a fluorescence-based assay using an artificial non-fluorogenic substrate for rFC. The rFC is activated in the presence of LPS and results in the cleavage of the artificial substrate and liberation of a fluorescent product. The rFC assay has sensitivity equivalent to the LAL test [24] and more significantly, is simpler to implement and therefore is more suited to LPS detection in the field than the traditional LAL assay.

Thus, the specific objectives of the current study are to (i) demonstrate that rFC assays can be performed in a field environment; (ii) demonstrate rFC assays can be used in the field to detect LPS in low biomass glacial samples and (iii) explore field-based sample processing protocols for the rFC assay. To achieve the study objectives the unique facilities at the low biomass glacier, Engabreen (Norwegian Arctic) were exploited, where the Svartisen Subglacial Laboratory allows direct access to pristine subglacial samples.

## 2. Materials and Methods

### 2.1. Field Site

Samples were collected from, and analysed at, Engabreen and its vicinity in November 2007, November 2008 and March 2009. Development of an in-field assay protocol was conducted during November 2007, and during November 2008 and March 2009 samples were collected for systematic analysis. Engabreen is a temperate outlet glacier draining the Svartisen Ice Cap, Northern Norway (66°41′N, 13°46′E). Facilities provided by the Norwegian Water Resources and Energy Directorate (NVE) allow direct access to pristine samples at the glacier bed via Svartisen Subglacial Laboratory [25]. Access to the glacier bed was through a series of tunnels drilled into the bedrock. These lead to locations where fresh subglacial meltwater and basal ice (amongst others types of samples) were collected. Pristine basal ice samples were collected from a temporary ice cave bored into the glacier using hot water in the research shaft. All in-field laboratory analyses were conducted in a field-laboratory housed within the subglacial rock tunnels.

### 2.2. General Materials, Methods and Shipping

All consumable items were freighted or hand carried to the field site. The assay reagents and fluorometer were hand carried to the field site where the rFC assay kit was transported in a cool box, with cool packs. The rFC kit was tested upon arrival during each field campaign to confirm usability.

Pipette tips and Eppendorf tubes were sterilised by autoclave, both prior to shipment to the field site and at the field site. Glassware and aluminium foil were depyrogenated by heating to 250 °C for 4 h prior to shipment and packaged in depyrogeneted foil. The water used to dilute standards and blanks was certified endotoxin free (supplied with rFC assay kit) and to minimise shipping, local subglacial water (filtered through 0.22 *μ*m pore cellulose membrane and autoclaved three times) was used to make buffer solutions needed in the additional sample processing protocols. All chemicals were obtained from Sigma-Aldrich (Poole, UK) unless otherwise stated.

### 2.3. Sample Collection and Handling

**Figure 1:**
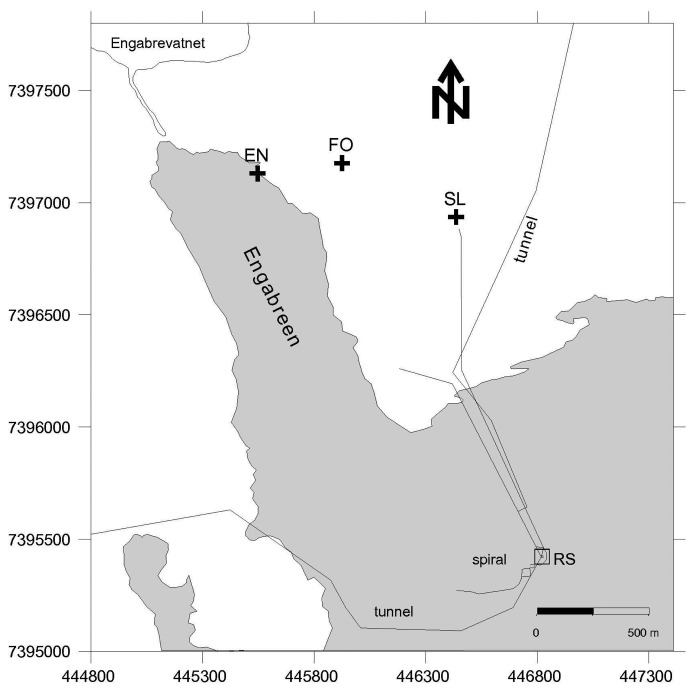
Map of Engabreen area with locations of the subaerial stream sampling sites and water access tunnels (black lines). EN—englacial stream; FO—forest stream; SL—shrubland stream and Engabrevatnet is the proglacial lake. The proglacial stream sample in 2008 was in the position same as EN (2009), the snow samples were collected from near the shrubland stream site, and the basal ice samples were collected from the research shaft (RS). The outline of the glacier is from 1995, but similar to November 2008 and March 2009.

Sediment, ice and meltwater samples were collected from the glacier snout and proglacial plain (subaerial samples), and subglacial environments (via the Svartisen Subglacial Laboratory), and were collected on different days during three one-week-long field campaigns from 19-27 November 2007, 19-26 November 2008 and 18-27 March 2009. In November 2008 and March 2009 a systematic sampling strategy was applied to collect equivalent samples, as far as practical, from both field campaigns. In November 2008 and March 2009 the samples clustered into three sample locations: subglacial meltwater collected from bedrock tunnels directly beneath the glacier; basal ice samples from the ice cave; and subaerial samples *i.e.*, external to the Svartisen Subglacial Laboratory and tunnels (Figure 1). The subglacial meltwater samples (coded MW 1 to 5) were collected from different locations along a ∼600 m tunnel (the Spiral Tunnel and Stream), sites MW 1 and 2 were under the centre of the glacier and site MW 5 was the closest to the glacier margin. The samples were collected from subglacial inlets in the tunnel roof (sites MW 1, 2 and 3) and from fast flowing portions of the meltwater stream (sites MW 4 and 5). Site MW 5 was at the confluence of an additional subglacial meltwater stream and associated tunnel close to the research shaft (RS in Figure 1). The Spiral Tunnel consists of purely subglacial meltwater and the additional stream mainly drains snowmelt and/or rainwater. Basal ice samples (coded BI 1 to 6) were collected along a transect, from ice in contact with bedrock into glacial ice, where the total perpendicular distance from the bedrock contact to the furthest bulk ice sample was **∼**5 m. BI 1 to 3 were debris rich ice samples, where BI 1 was in contact with the bedrock and BI 3 was near the contact with the glacial ice. BI 3 to 6 were glacial ice (or sediment poor ice), where BI 4 was near the contact with debris rich ice and BI 6 was furthest from the contact. The external samples consisted of snow, and water from englacial, proglacial, and forest streams (locations in Figure 1).

All water samples were collected in autoclaved 250 mL Beckman bottles (Beckman, USA), or in sterile 1.5 mL Eppendorf tubes and returned to the field-laboratory for processing and analysis. All bottles were rinsed at least three times with sample water before being filled. Sediment samples were collected in 50 mL sterile centrifuge tubes. Snow samples were placed into sterile sample bags using gloves wiped in 96% ethanol. The basal ice samples were collected from the ice cave in the research shaft. In 2007 and 2008 this was conducted using an ice axe or chisel, previously wiped with 96% ethanol. In 2009 the samples were extracted using a chainsaw. Ice was wrapped in depyrogenated foil within the ice cave, moved to the field-laboratory and melted overnight in clean autoclave bags or sterile sample bags. The surface of the chainsaw collected samples was washed with ethanol and then with warm autoclaved water to remove surface contaminants. All samples were assayed within 24 h of collection or melting.

### 2.4. Recombinant Factor C Assay

The Cambrex PyroGene^®^ recombinant Factor C (rFC) endotoxin detection system (product code 50-658U, Lonza, Slough, UK) was used for the study. Each assay comprised a 50 *μ*L sample mixed with 50 *μ*L of the rFC working reagent (proenzyme, fluorescent substrate and buffer), made up as per manufacturer guidelines. Assays were incubated at ambient field-laboratory temperature for 3 h, where the temperature varied between +17 °C and +21 °C. Fluorescence measurements were made using a handheld *Pico*fluor fluorometer (Turner BioSystems, CA, USA) at time 0, and then after 1, 2 and 3 h incubation time in November 2007 and every 30 min for 3 h in November 2008 and March 2009. Readout of the fluorometer was in Relative Fluorescence Units (RFU). To allow for the small reagent volumes indicated in the manufacturer guidelines, the Picofluor was used with a mini-cell adapter and 70-250 *μ*L borosilicate glass cuvettes (product code WU-13095-56, Turner BioSystems, Cole Parmer, London, UK). All samples were assayed in triplicate.

The endotoxin (LPS) standards supplied with the rFC kit are quoted in endotoxin units (EU), which is a measure of the biological activity of LPS. LPS standards of 10, 1, 0.1 and 0 EU·ml^−1^, were tested for each batch of working reagent.

An alternative to the standard data analysis described in the rFC assay kit was implemented in the current study. This was implemented to allow for (i) non-standard and varying assay incubation temperatures and (ii) uncertainty in the time evolution of the assay under in-field conditions. The implemented analysis comprised of plotting the fluorescence intensity as a function of assay time. For the majority of samples analysed, when using an appropriate incubation time, this resulted in a linear relationship. Therefore for all samples and standards analysed, a linear function was fitted to the data (between time zero and the end of the incubation period) using the least squares method. R**^2^** values were calculated for each assay to assess the appropriateness of the linear model for each analysis. The gradient of the fitted function (termed assay response, in RFU•min^−1^) was used for further data analysis. LPS concentrations in samples were quantified from calibration data sets produced from the LPS standards, where a linear function was fitted to a log-log plot of assay standards and assay responses. The lower limit of detection of LPS was defined as assay response of the appropriate blank plus three times the standard deviation of the assay response of the blank.

### 2.5. Sample Processing Protocols

A sample processing protocol was defined that reflected a desire to minimise field-based sample processing (minimal processing protocol). To assess whether more involved, but still field deployable, sample processing would improve analytical performance (*i.e.*, decreased lower limits of detection), two additional sample processing protocols were tested (additional protocol A and additional protocol B). Three sample types were considered: water, ice and sediment.

#### 2.5.1. Direct Analysis (Minimal Processing Protocol)

The water samples were directly assayed. For sediment samples an equal volume of LPS free water was added to the sediment, sample was shaken vigorously for 10 s and immediately a liquid aliquot including suspended particulates was taken and assayed. Ice samples were melted at ambient laboratory temperature and moved to +3 °C storage once melted. The melted ice samples were then agitated to suspend fine particulate and a representative liquid aliquot was assayed.

#### 2.5.2. Boiling in Tris EDTA Buffer (Additional Protocol A)

For water samples, 490 mL of sample was filtered through an autoclaved 47 mm diameter, 0.22 *μ*m pore cellulose membrane (Millipore, FDR-293-050F, Fisher, Loughborough, UK) using a polysulfone filter unit and hand vacuum pump. The filter membrane was folded and placed into a 1.5 mL Eppendorf tube and physically broken, using flamed metal tweezers, to improve subsequent access to the extraction buffer. To this, 1 mL of boiling 100 mM Tris, 4 mM EDTA extraction buffer was added, followed by incubation at 100 °C for one minute. Tubes were cooled and stored at +3 °C and representative liquid aliquots assayed within 24 h. Due to the high volume of sample filtered, this extraction protocol was appropriate for water samples with low sediment concentrations. Thus, ice and sediment samples were not used for this method.

#### 2.5.3. Ultrasonic Treated at Ambient Temperature in Tris Saline Buffer (Additional Protocol B)

This processing protocol was most suited to sediment and sediment rich ice samples, but water samples were also tested. For water and melted ice 15 mL of sample and for sediment 2 g of sample was added to 15 mL of 25 mM Tris, 100 mM sodium chloride buffer in 50 mL centrifuge tubes. The tubes were subjected to three sets of 10 s duration in a small ‘hobby’ sonicator (James Products Ltd, Ultra 6000WS, product code A49FW, Maplin Electronics, Rotherham, UK). Solutions were cooled in an ice bath between each sonication treatment. Samples were agitated and then a representative liquid aliquot was assayed.

## 3. Results

### 3.1. Adaptation of rFC Assay Protocol for in-Field Analysis

The manufacturer's protocol for performing the rFC assay state a requirement for incubation of the mixed sample and assay working reagent at +37 °C in a fluorescence microplate reader, and that fluorescent intensity measurements should be taken at time zero and after one hour incubation; the difference between the two values should be used to calculate LPS concentration. The logistics of establishing the rFC assay at a glacial field site precluded the use of a standard laboratory specification fluorescent microplate reader with incubation temperature control. A non-temperature controlled handheld fluorometer compatible with reagent volumes needed for the rFC assay and easily transportable to the field site was identified and used. Incubation was conducted in a field laboratory with an internal ambient temperature in the range of +17 °C and +21 °C. This adaptation to the method necessitated some modifications to the manufacturer's assay instructions, and these were studied prior to the field campaigns.

**Figure 2:**
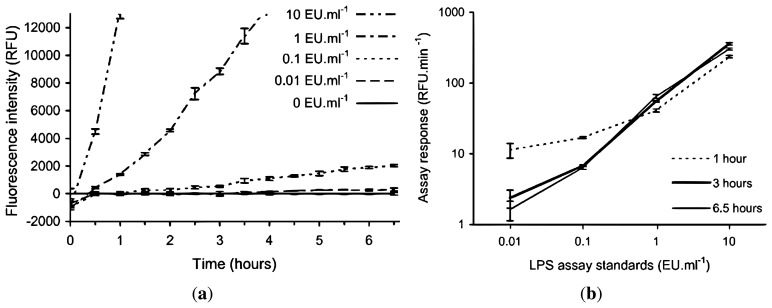
Effect of non-standard incubation temperature on the rFC assay using LPS standards to determine modified rFC assay protocol for field deployment. The 10, 1, 0.1 and 0.01 EU·mL^−1^ standards are normalised by subtraction of the 0 EU·mL^−1^ standard. (**a**) Time evolution of the endotoxin standards, *y*-axis is truncated, the 10 EU·mL^−1^ standard starts to plateau after 4 h and 1 EU·mL^−1^ standard continues to increase linearly (RFU = relative fluorescence units); (**b**) Calibration curves at 1, 3 and 6.5 h (*i.e.*, assay response between t = 0 and time of interest). Error bars represent ±1 SD from triplicates.

Due to the expected lower and fluctuating incubation temperatures encountered in the field laboratory, a lower rate of fluorescence intensity development for the assay was anticipated, therefore requiring a longer incubation period to develop a suitable signal for a given LPS concentration. In order to test this hypothesis and to determine the required length of incubation time, the rFC assay was conducted with LPS standards (10, 1, 0.1, 0.01 and 0 EU·mL^−1^) in an institutional laboratory with an ambient temperature of +20 °C to +22 °C over an incubation period of 6.5 h. Figure 2 shows the fluorescent intensities recorded at half hourly intervals using the handheld fluorometer, and the corresponding calibration curves if the assay was stopped after 1, 3 and 6.5 h. The 10 EU·mL^−1^ LPS standard starts to plateau after 4 h (not shown in Figure 2(a)), but the 1 EU·mL^−1^ LPS standard continues to increase in intensity linearly (by visual inspection) during this time. Figure 2(b) shows the assay response for the 0 and 0.1 EU·mL^−1^ LPS standards is greatest when the incubation time is limited to one hour, which is an artefact of the low RFU values for each standard at time zero, as seen in Figure 2(a). The lower time zero data points have a greater effect on the standards when the incubation time is short. Figure 2(a) is normalised to the blank, the standard deviations (SD) of triplicates of the standards including the blank are included to demonstrate the ability to discriminate between the standards.

The results in Figure 2(a) show that if the manufacturer's recommended one hour incubation time was to be followed with an incubation temperature similar to the expected field laboratory conditions, then discrimination between the 10 and 1 EU·mL^−1^ standards and between these and 0 EU·mL^−1^ standard was possible but, no significant discrimination between 0.1 and 0 EU·mL^−1^ standards could be made. When the incubation time was extended to 3 h, then discrimination among 10, 1 and 0.1 EU·mL^−1^ standards and between these and 0 EU·mL^−1^ standard was now possible. Extending the incubation time beyond 3 h does improve the separation between the 0.01 and 0 EU·mL^−1^ standards however it is only a minor increase. Also, extending incubation time to 3 h is still practical for field analysis.

Since the in-field assay response of the rFC assay and its interaction with environmental samples was unknown prior to field deployment, it was considered prudent to collect fluorescence intensity data at a minimum of hourly intervals during the 3 h incubation period in addition to the initial and final data points. This would allow confirmation of the expected time evolution of the assay response as determined in the laboratory (Figure 2(a)) and any significant deviation from this was identified by calculation of Coefficient of Determination (R^2^) values.

### 3.2. Testing of Additional Sample Processing Protocols for Glacial Samples for Use in rFC Assays

To assess the effect of minimal sample processing (*i.e.*, minimum amount of processing required to obtain liquid samples, e.g., melting ice) compared to protocols with additional sample processing for LPS detection, we conducted a range of tests on field samples during the November 2007 field campaign. The additional sample processing protocols comprised of filtration then boiling of the filter membrane in Tris EDTA buffer (additional protocol A) and ultrasonic treatment at ambient temperature in Tris saline buffer (additional protocol B). For each additional sample processing protocol, a set of six different samples was analysed and compared to the same sample set that underwent the minimal sample processing protocol. Due to operational reasons, different sample sets for additional protocol A and B were used.

Table 1 shows the assay responses (the gradients of the assumed linear fluorescence intensity change as a function of time for a given rFC assay) and R^2^ values for the samples analysed using the two additional sample processing protocols and the equivalent analyses using the minimal sample processing protocol.

**Table 1:** Assessment of two additional sample processing protocols for LPS determination in glacial samples when compared to the use of the minimal sample processing protocol. Note that all assay responses are derived from samples analysed in triplicate.

**Sample description**	**Assay response (RFU·min^−1^)**		**R^2^**	
	
	**Default protocol**	**Additional protocol**	**Default protocol**	**Additional protocol**
**Boiling in Tris EDTA buffer (additional protocol A)**				
Blank	0.74	0.74	0.240	0.215
Pro-glacial stream (snout)	14.31	−0.98	0.946	0.312
Pro-glacial stream (side)	19.99	−0.84	0.975	0.495
Periglacial iron-rich pool	1.83	1.22	0.626	0.658
Subaerial forest stream	14.82	12.49	0.994	0.969
Subaerial scrubland stream	4.37	0.23	0.962	0.165
Subglacial meltwater 4 (day 4)	5.57	−0.72	0.856	0.322
**Ultrasonic treated at ambient temperature in Tris saline buffer (additional protocol B)**				
Blank	−0.66	−0.92	0.914	0.592
Subglacial meltwater 5 (day 6)	1.22	−0.53	0.657	0.962
Bedrock tunnel sediment (site MW 5)	−0.32	10.47	0.916	0.981
Bedrock tunnel water (stagnant pool)	80.89	8.99	0.958	0.979
Bedrock tunnel sediment (stagnant pool)	26.25	−3.71	0.998	0.972
Glacial basal ice	−5.29	−2.25	0.977	0.606
Groundwater (borehole)	16.62	0.21	0.950	0.057

The results in Table 1 show that three of the seven samples (including the blank) processed using additional protocol A had a similar assay response to the minimal processing protocol (*blank*, *subaerial iron-rich pool* and *subaerial forest stream*). However, for the remaining four samples, the assay response of additional protocol A was significantly lower than the assay response of the minimal processing protocol, and was near or below zero (0.23 to −0.98 RFU·min^−1^). Comparing the assay responses of the minimal processing protocol and additional protocol B, the assay responses were lower for additional protocol B than for the minimal protocol in all samples (including the blank) except for the *bedrock tunnel sediment* sample where it was higher. Table 1 shows neither the assay responses nor the R^2^ values observed for both of the additional sample processing protocols showed a consistent improvement in comparison to the minimal sample processing protocol. Hence for the November 2008 and March 2009 field campaigns, the minimal sample processing protocol was used exclusively.

### 3.3. In-Field Use of rFC Assay to Detect LPS in Glacial Samples with Minimal Sample Processing

**Figure 3:**
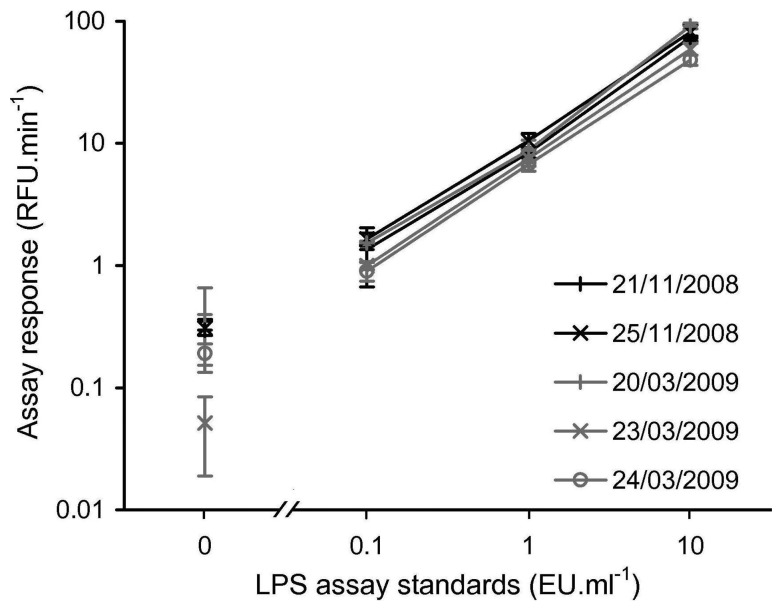
Calibration curves produced on the five days during the 2008 and 2009 field campaigns *i.e.*, rFC assay response for 10, 1, 0.1 and 0 EU·ml^−1^ standards. Error bars indicate ±1 SD of the assay response from triplicates. Note log scale and broken *x*-axis to allow for visualisation of the 0 EU·ml^−1^ standards.

To confirm that the in-field rFC assay protocol was a repeatable technique when conducted in the field, LPS assay standards of 10, 1, 0.1 and 0 EU·ml^−1^ were tested on each day the assay was completed during the November 2008 and March 2009 field campaigns, producing what is referred to as a LPS calibration set for each day. Figure 3 shows that within each LPS calibration set, there is a clear positive relationship between LPS concentration and rFC assay response, and that the individual LPS assay concentration standards are easily discriminated. Furthermore, for a given LPS concentration standard, the spread of assay responses between the five different LPS calibration sets is acceptable.

**Figure 4:**
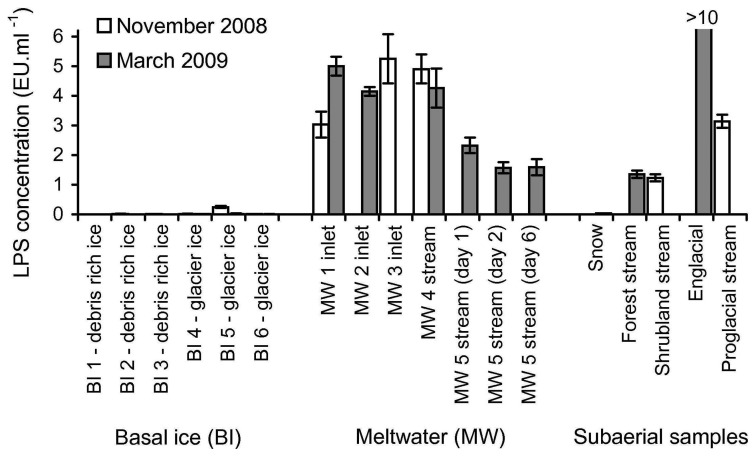
Determination of LPS levels in 27 glacial samples using the rFC assay. Details of the sampling sites are described in Section 2.3. All assays were performed with no significant sample processing (minimal processing protocol). LPS concentration was quantified by using the daily LPS calibration sets, assuming a linear interpolation from a log-log plot of LPS standards (10, 1 and 0.1 EU·ml^−1^) and the assay responses. The *y*-axis (LPS concentration) has been truncated (*i.e.*, the value for 2009 englacial water is clipped) to enable better visualisation of the relationship of the LPS concentration values of the other samples. Error bars are ±1 SD of assay responses from triplicates.

To demonstrate that the rFC assay could detect LPS in a variety of glacial samples in the field, 27 samples from various subglacial and subaerial locations (locations described in Section 2.3) were tested for the presence of LPS. Figure 4 shows LPS concentrations measured in the samples assayed.

The results in Figure 4 show that LPS was detected in 16 of 27 samples (*i.e.*, sample assay response was greater than the assay response of the blank, plus three times the standard deviation of the assay response of the blank). The presence of LPS was detected in all nine of the subglacial meltwater samples, in five of the six external samples (excluding sample *2008 snow*), and in only two of the twelve basal ice samples (samples from site BI 5 from both campaigns). Using the LPS calibration sets (10 to 0.1 EU·ml^−1^ standards), LPS concentration could be quantified in 13 of the 16 samples with detectable LPS. Of the three samples where LPS was detectable but not quantifiable, two had an LPS concentration <0.1 EU·ml^−1^ (samples *2009 snow* and *2009 BI 5*) and one had an LPS concentration >10 EU·ml^−1^ (sample *2009 englacial*). Figure 4 shows there is two to three fold variability in the nine subglacial meltwater samples (3.56 ± 1.46 EU·ml^−1^). However the three samples collected from site MW 5 (1.83 ± 0.43 EU·ml^−1^) are consistently lower than the six samples from sites MW 1 to 4 (4.43 ± 0.81 EU·ml^−1^). The subaerial samples show the most variability, including samples below 0.1 EU·ml^−1^ and above 10 EU·ml^−1^. The R^2^ values for the 16 samples with detectable LPS ranged from 0.978 to 0.998, whereas the R^2^ values of the remaining 11 samples range between 0.073 and 0.997 (data not shown).

## 4. Discussion

The results in Figure 2 show that, in order to use the rFC assay in the field under ambient conditions, modifications to the recommended manufacturer's protocol were necessary. Firstly, the manufacturer's protocol required an incubation temperature of +37 °C. Since the laboratory at our glacier field site was between +17 °C and +21 °C we developed and demonstrated a modified assay protocol that involved a longer incubation period—increased from a manufacturer's stated time of 1 h to 3 h. This allowed sufficient assay signal to be developed to discriminate LPS concentrations over two orders of magnitude. The increased incubation period can be accounted for by the expected reduced reaction rate at lower temperatures. The assay of a LPS calibration standard data set in parallel with all sample analyses enabled for the correction of any variation in the assay reaction rate as a function of temperature and enzyme activity, which can be modified by non standard storage conditions such as those encountered during transportation. We note that under colder ambient conditions in many glacial environments, the incubation time may need to be increased above 3 h. Further studies are required to define appropriate incubation periods for lower incubation temperatures. At very low temperatures, field deployable incubation equipment such as a small dry block heater may be required. A second modification was due to the manufacturer's stated assay protocol that required only fluorescent measurements at the beginning and end of the incubation period. Due to the uncertainty surrounding the effect of sample matrix on the time evolution of the assay response, the data set was increased by the collection of additional fluorescent measurements during the incubation period. For the November 2008 and March 2009 samples, unexpected, non-linear time evolution of the assay responses (meaning R^2^ values < 0.95) was observed only in samples where LPS was not detectable.

As the manufacture's application of the rFC assay is to test human and animal parenteral drugs, biological products and medical devices, the intended sample types for the commercial assay are considerably different to glacially relevant samples. Therefore two sample processing methods comprising filtration (concentration) and/or cell lysis (to liberate LPS into a more available form) were considered with the intention of decreasing the lower limit of detection and, additional to the minimal processing protocol needed to have samples as a liquid. The two additional processing methods were chosen as they were also being considered as sample processing methods for other analysis techniques being used in the same field campaign (November 2007). Additional protocol A consisted of filtration of a water sample to concentrate microbial cells on the filter membrane, followed by boiling the membrane in Tris EDTA buffer. Despite a 490-fold concentration step involved (see Section 2.5.2), no improved LPS detection was observed compared to the minimal protocol. The assumed reason for this was the strong adsorption of LPS to the cellulose filter paper used in the protocol [26]. Additional protocol B consisted of ultrasonic treatment at ambient temperature in Tris saline buffer, with no prior filtration. The ultrasonic treatment was intended to lyse microbial cells and thus liberate LPS into a more accessible form for the rFC assay reagents. No consistent improvement in LPS detection was observed, which is probably due to a number of factors including that the bacterial cells were likely to have been lysed by the rFC reagents releasing LPS, as observed with the LAL assay [27], and the power of the ‘hobby’ sonicator used in this study was too low to disrupt significant additional Gram-negative cells. Hence during the November 2008 and March 2009 field campaigns only the minimal sample processing protocol was used. It is anticipated that further study will enable decreased lower limits of detection by sample processing protocols with appropriate consideration of material choices, *i.e.*, to avoid LPS adsorption.

The use of LAL and rFC assays for the detection of LPS in pharmaceutical and other established applications is considered to have very low, lower limit of detection under laboratory conditions. Typical values reported under these conditions are 0.01 EU·ml^−1^ which represents about ∼10^2^ cells·ml^−1^, where 10 EU represents ∼10^5^ cells [21,28]. The rFC assay's low, lower limit of detection and the presence of environmental microorganisms make the rFC assay prone to contamination. Hence, the use of rFC assays in field environments may lead to levels of contamination that severely compromise the analytical utility of the technique. Within the current study, it was only practical to apply standard good microbiological practice due to the logistical issues of working at a remote field site (e.g., ethanol wiping gloves and other sampling equipment and which is not expected to be an efficient method for removing LPS). To confirm whether contamination was a significant issue, the analysis of a blank sample (consisting of certified LPS free water) was included in each LPS calibration set. The assay responses of the blank samples were between 0.05 and 0.39 RFU•min^−1^ which is below the range of the lowest standard used in the field (assay response of 0.1 EU·ml^−1^ 0.90 to 1.65 RFU•min^−1^). As all the sample blanks returned an assay response less than the 0.1 EU·ml^−1^ standard the rFC assay could be conducted in the Engabreen field environment without significant contamination. In summary, in-field contamination does not represent a fundamental problem for field based implementation of the rFC assay, however to confirm this appropriate contamination controls should be completed with each analysis batch.

To demonstrate the in-field detection of LPS, a suite of contrasting samples was collected at or near Engabreen over two field campaigns (November 2008 and March 2009), and co-assayed with standards to allow for quantification (Figure 3 and Figure 4). The detection of LPS in 16 of the 27 subglacial and subaerial samples enabled the basic objective of demonstrating rFC assay as an in-field technique to be achieved.

For the initial interpretation of the preceding LPS data in the context of the microbiology of the Engabreen system, the 27 samples can be considered in three clusters; (i) subaerial samples; (ii) subglacial meltwater and (iii) basal ice. A systematic sampling strategy was applied to collect equivalent samples from both field campaigns. Unfortunately the practicalities of collecting equivalent samples in these two field campaigns and the range of samples that returned detectable LPS limit any inferences that can be made on the differences between the spring and autumn behaviour of Engabreen.

A range of subaerial sampling sites was chosen to contextualise the data from the subglacial environment. These comprised fresh snow, and waters from shrubland, forest, proglacial, and englacial streams. The fresh snow samples (both *2008 snow* and *2009 snow*) exhibited either no or very low levels of detectable LPS (<0.1 EU-mL^−1^) as would be expected as these samples had very low particulate concentration (from visual inspection of the filter paper), which has been shown to correlate to bacterial concentration in snow [29]. Waters from shrubland and forest streams had similar levels of detectable LPS (0.99 and 1.00 EU-mL^−1^). This is unexpectedly low in comparison to the subglacial samples, as it would be expected that the environment around the glacier would have a higher microbial concentration and hence LPS concentration [8,30]. To fully explain these results require further investigations into the proportion of the Gram-negative bacterial populations in these systems and the hydrological controls *i.e.*, the relative contributions of snowmelt and rainwater. The proglacial stream sample was expected to be predominately fed by subglacial meltwater and therefore would be expected to have had LPS values similar to the subglacial meltwater samples. For the single proglacial stream sample assayed, the detected level of LPS (3.13 EU-mL^−1^) was within the spread of values obtained for the subglacial meltwaters. Lastly the englacial stream sample produced the highest LPS value (>10 EU-mL^−1^) of all the samples during both field campaigns. Whilst unexpectedly high, englacial streams are ill-defined as they can be draining local features, including snowpacks, where bacterial numbers could be much higher than fresh snow, if sediment concentration increases [29].

The sites for the subglacial meltwater samples fall into two groups. The LPS levels from all samples from sites MW 1 to MW 4 (the Spiral Tunnel) are similar (4.43 ± 0.81 EU-mL^−1^ and n = 6) and higher than all samples from site MW 5, which is at the confluence of two streams (1.83 ± 0.43 EU-mL^−1^ and n = 3). The interpretation of this is the LPS load in the additional stream contains significantly less LPS than in the Spiral Tunnel Stream and which accounts for a dilution effect at the confluence. This appears to be a reasonable suggestion as the two streams are distinct in terms of source, location and size of their catchment areas (described in Section 2.3). The high concentrations of LPS in these meltwaters, equivalent to approximately 2 to 4 × 10^4^ Gram-negative bacteria per mL, are consistent with other investigations that show significant microbial populations in subglacial environments [5,7].

Six of the melted basal ice samples were considered to be glacier ice *i.e.*, sediment poor ice away from the influence of bedrock erosion (samples from sites BI 4 to BI 6 defined visually) and six samples to be debris rich ice (samples from sites BI 1 to BI 3 defined visually). LPS was detected using the rFC assay in only two of the twelve samples (samples *2008 BI 5* and *2009 BI 5*), and LPS concentration was quantifiable in one of these (sample *2008 BI 5*). Both these samples were glacier ice. There are two potential explanations for non-detection of LPS in the majority of the basal ice samples; the concentration of LPS is below the detection limit of the rFC assay or there is interference with the rFC assay from the sample. The concentration of microorganisms in glacier ice is known to be low (from 10^2^ to 10^3^ cells·ml^−1^ Midre Lovenbreen, Spitsbergen [31]). The lack of detection of LPS in the majority of the glacial samples is consistent with this observation as it suggests that LPS concentration approaches the lower limit of detection that can be obtained with the in-field rFC assay in this type of sample. It was observed that the sample with quantifiable LPS concentration (2008 BI 5) had some minor sediment inclusions, although substantially less than any of the debris rich ice samples, thus increasing potential concentration of microorganisms as the majority are associated with sediment load [5,32,33]. The association of microorganisms with sediment suggests that debris rich ice samples should contain a higher concentration of microorganisms than 2008 BI 5, and hence an easily detectable concentration of LPS. Given that none of the debris rich samples gave a detectable LPS signal, we infer the non-determination of LPS in the debris rich ice (samples from sites BI 1 to BI 3) reflects interference of the sediment with the rFC assay. Detection of LPS in some of the glacier ice (but not sediment free) samples suggests that the effect of this interference scales with sediment concentration. The source of this interference could be due to one or more of the three following factors: adsorption of LPS by the sediment, the sediment acting as a fluorescence quencher or the sediment denatures rFC enzyme. Possible methods for reducing the interference involve (i) removal of the sediment (but this could also remove the cells); (ii) reduce the sediment concentration or (iii) increase the availability of LPS. These methods could be achieved by one or more of the following; cell lysis or, dilution, filtration or centrifugation of sample prior to analysis.

In conclusion we have demonstrated for the first time, in a glacial environment, the in-field detection of LPS, a proxy for Gram-negative bacteria, and thus the presence of extant life. This was conducted using a recently commercialised bioassay kit (rFC assay) together with a modified assay protocol and a portable fluorometer to enable use. The suitability for in-field detection of LPS by the rFC assay included the analysis of samples directly without the need for significant sample processing. A diverse range of glacial sample types was tested for LPS including basal ice, subglacial meltwater and samples external to the subglacial environment. LPS was detected successfully in each of these sample groups. The limitations of the current in-field rFC assay protocol were encountered only with very low biomass samples (∼10^3^ cells·ml^−1^, where 10 EU is ∼10^5^ cells [21,28]), and samples with a significant fraction of fine particulates. Hence further investigation into sample processing approaches will be required to optimise the rFC assay for these sample types. The modified rFC assay, as demonstrated by the detection of LPS in glacial samples, has potential applications in other environmental low biomass samples, as it offers (i) a new technique for the in-field detection of extant life using LPS as a proxy and (ii) the ability to determine additional microbial community structure by the detection of LPS as a proxy of Gram-negative bacteria when employed in conjunction with other less specific biomarker techniques, e.g., ATP bioluminescence or fluorescent cell counts.
